# fMRI Reveals Mitigation of Cerebrovascular Dysfunction by Bradykinin Receptors 1 and 2 Inhibitor Noscapine in a Mouse Model of Cerebral Amyloidosis

**DOI:** 10.3389/fnagi.2019.00027

**Published:** 2019-02-15

**Authors:** Ruiqing Ni, Diana Rita Kindler, Rebecca Waag, Marie Rouault, Priyanka Ravikumar, Roger Nitsch, Markus Rudin, Giovanni G. Camici, Luca Liberale, Luka Kulic, Jan Klohs

**Affiliations:** ^1^Institute for Biomedical Engineering, University of Zurich and ETH Zurich, Zürich, Switzerland; ^2^Zurich Neuroscience Center, Zürich, Switzerland; ^3^Institute for Regenerative Medicine, University of Zurich, Zürich, Switzerland; ^4^Center for Molecular Cardiology, University of Zurich, Zürich, Switzerland; ^5^Department of Internal Medicine, University of Genoa, Genoa, Italy

**Keywords:** Alzheimer’s disease, beta-amyloid, bradykinin receptor, cerebral blood volume, cerebrovascular dysfunction, magnetic resonance imaging, perfusion imaging

## Abstract

Functional magnetic resonance imaging (fMRI) techniques can be used to assess cerebrovascular dysfunction in Alzheimer’s disease, an important and early contributor to pathology. We hypothesized that bradykinin receptor inhibition alleviates the vascular dysfunction in a transgenic arcAβ mouse model of cerebral amyloidosis and that fMRI techniques can be used to monitor the treatment response. Transgenic arcAβ mice, and non-transgenic littermates of 14 months-of-age were either treated with the bradykinin receptors 1 and 2 blocker noscapine or received normal drinking water as control over 3 months (*n* = 8–11/group) and all mice were assessed using fMRI at the end of the treatment period. Perfusion MRI using an arterial spin labeling technique showed regional hypoperfusion in arcAβ compared to non-transgenic controls, which was alleviated by noscapine treatment. Similarly, measuring cerebral blood volume changes upon pharmacological stimulation using vessel dilator acetazolamide revealed recovery of regional impairment of cerebral vascular reactivity in arcAβ mice upon noscapine treatment. In addition, we assessed with immunohistochemistry beta-amyloid (Aβ) and inflammation levels in brain sections. Immunohistological stainings for Aβ deposition (6E10) and related microgliosis (Iba1) in the cortex and hippocampus were found comparable between noscapine-treated and untreated arcAβ mice. In addition, levels of soluble and insoluble Aβ_38_, Aβ_40_, Aβ_42_ were found to be similar in brain tissue homogenates of noscapine-treated and untreated arcAβ mice using electro-chemiluminescent based immunoassay. In summary, bradykinin receptors blockade recovered cerebral vascular dysfunction in a mouse model of cerebral amyloidosis. fMRI methods revealed the functional deficit in disease condition and were useful tools to monitor the treatment response.

## Introduction

Alzheimer’s disease (AD) is the most common form of dementia and represents a complex and multi-factorial disorder. Hallmarks of the disease are the accumulation of abnormal beta-amyloid (Aβ) and neurofibrillary tangles composed of hyperphosphorylated microtubule-associated tau protein, leading to neurodegeneration (Braak and Braak, 1991). Misfolded Aβ and tau protein bind to receptors on microglia and astroglia, and trigger innate immune responses, which contribute to disease progression and severity (Heneka et al., 2015). In addition, alterations in the density and morphology of cerebral vasculature, a regional reduction of cerebral blood flow (CBF) and an impaired cerebral vascular reactivity (CVR) have been reported in patients with AD and mild cognitive impairment (de la Torre, 2004; Kisler et al., 2017), and have been implicated to contribute to the cognitive impairment.

Several neuroimaging techniques have been developed to assess features of AD pathology in patients and animal models of the disease. The quest is to improve the diagnosis of the disease, to assess the disease stage, to select patients for treatment and to monitor the response to therapy. Among them, magnetic resonance imaging (MRI) has been used to detect degenerative changes in patients with AD (Frisoni et al., 2010). More recently, functional MRI (fMRI) techniques have been implemented to evaluate cerebrovascular abnormality in patients with AD, which has been shown to occur early during the disease course (Iturria-Medina et al., 2016). These comprise regional changes in CBF (Maier et al., 2014) and microvessel density (Klohs et al., 2012), disturbances of blood–brain barrier integrity (Montagne et al., 2017), impairment of vascular reactivity (Stoppe et al., 1995; Mueggler et al., 2002; Princz-Kranz et al., 2010; Dumas et al., 2012), and vascular remodeling (Dumas et al., 2012; Klohs et al., 2016).

The kallikrein-kinin system has been implicated as an important pathophysiological mediator of cerebral vascular dysfunction, neuroinflammation and Aβ pathology in AD (Schmaier, 2016; Nokkari et al., 2018). Kinins, in particular bradykinin, are pro-inflammatory mediators with a range of physiological effects in the periphery, which have also been described in the central nervous system (Couture et al., 2000). Bradykinin, lysine-bradykinin, and bradykinin degradation products act through the activation of two G protein-coupled bradykinin receptors (BRs): B_1_R and B_2_R. Pharmacological antagonism or genetic deletion of B_1_R (Prediger et al., 2008; Lacoste et al., 2013; Passos et al., 2013; Asraf et al., 2016) and B_2_R (Bicca et al., 2015; Caetano et al., 2015) have been shown to alleviate the cognitive deficits in Aβ-injected or transgenic AD animal models.

In the present study, we hypothesized that chronic oral treatment with the phthalideisoquinoline alkaloid noscapine, a B_1_R and B_2_R antagonist (Landen et al., 2004), ameliorates the CBF and CVR abnormalities in arcAβ mouse model of amyloidosis (Merlini et al., 2011). We investigated the effect of BR blockade by noscapine on CBF and CVR in arcAβ mice using fMRI techniques. In addition, we studied the effects of noscapine treatment on Aβ deposits and microglia by immunohistochemical and biochemical analysis.

## Materials and Methods

### Animals

All experiments were performed in accordance with the Swiss Federal Act on Animal Protection and were approved by the Cantonal Veterinary Office Zurich (Permit Number: 90-2016). All procedures fulfilled the ARRIVE guidelines on reporting animal experiments. ArcAβ transgenic mice, with human APP695 transgene containing the Swedish (K670N/M671L) and Arctic (E693G) mutation under the control of the prion protein promoter (Knobloch et al., 2007), were used. Animals were kept in a temperature-controlled room in individually ventilated cages under 12 h light/dark cycle and access to pelleted food and water were provided *ad libitum*. Paper tissue was given as environmental enrichment. A sample size of *n* = 7 per group was calculated for the primary end point CBF, a fixed effect omnibus, one-way ANOVA with four groups, and an effect size *f* = 0.72, α = 0.05 and β = 0.2. Consequently, group sizes *n* > 7 were used (G^∗^power, University of Düsseldorf, Germany).

### Measurement of Plasma Bradykinin Levels

Six arcAβ and seven non-transgenic littermates (NTLs) of 17 months-of-age were used. Blood from the vena cava of the mice was collected into Eppendorf tubes filled with 50 μl 0.5 M ethylenediaminetetraacetic acid (EDTA, Sigma-Aldrich GmbH, Switzerland) and centrifuged at 4°C, 1000 *g* for 15 min. The blood : EDTA volume ratio was approximately 8:1. The plasma supernatants were collected as 50 μl aliquots, frozen and kept at -80°C, until use. The enzyme immunoassay (RayBiotech, Norcross, GA, United States) was used for bradykinin level measurement. A known concentration of biotinylated bradykinin was spiked into samples and standards, which were then added to wells. Here, biotinylated-bradykinin competes with the endogenous bradykinin in plasma for binding to the monoclonal anti-bradykinin antibody immobilized on the wall. After color development reaction, the intensity of the colorimetric signal was directly proportional to the amount of biotinylated bradykinin captured by the antibody, which inversely correlated to the amount of endogenous peptide in the sample or standard. A standard curve for the quantification of bradykinin concentration in the samples was generated. The lower detection limit of the assay was 1.4 ng/ml bradykinin.

### Study Design Noscapine Treatment

A flowchart of the design for the noscapine treatment study is shown in Supplementary Figure 1. Twenty arcAβ and 17 NTLs of both genders at approximately 14 months-of-age (at the start of the study) were used for the 3 months treatment study (Table 1). Animals of both genders were randomly allocated to experimental groups. Experimenters were blinded during data acquisition and analysis. Mice in the treatment arm were supplied with noscapine (Sigma-Aldrich GmbH, Switzerland) in acidified drinking water for 3 months before the first measurement. Noscapine (3 g/l) was dissolved in double distilled water, adjusted to pH 3.5 (Sigma-Aldrich GmbH, Switzerland). Prior to the treatment study, high performance liquid chromatography was performed to measure the stability of noscapine in acidified double distilled water (pH 3.5), where 94 and 70% of noscapine were detectable after 24 h and 2 days, respectively. Therefore, we prepared fresh noscapine solution (pH 3.5) daily and protected it from light to ensure stability. For the control group, double distilled water was provided. All animals were weighed once per week during the study. Blood pressure of the mice were assessed non-invasively at the end of the treatment by tail-cuff using CODA monitor (Kent Scientific, Corp., Torrington, CT, United States). Systolic and diastolic blood pressures of each mice were assessed consecutively twenty times, and were averaged (Table 1).

**Table 1:** Demographic information, weight, and blood pressure of mice.

Group	NTL	NTL noscapine	arcAβ	arcAβ noscapine
*N*	8	9	9	11
Female/male	4/4	7/2	5/4	4/7
Age (months)^∗^	16.6 ± 0.3	16.8 ± 0.4	16.6 ± 0.4	16.6 ± 0.4
Weight before treatment (g)	31.5 ± 4.3	28.1 ± 3.8	26.8 ± 4.7	29.1 ± 3.1
Weight end of treatment (g)	32.5 ± 4.6	28.3 ± 2.6	28.1 ± 4.4	29.3 ± 3.6
Systolic blood pressure (bpm)	86.3 ± 24.8	117.4 ± 16.0	107.8 ± 36.4	108.2 ± 22.6
Diastolic blood pressure (bpm)	61.8 ± 29.0	76.2 ± 11.9	75.1 ± 43.1	76.7 ± 24.7

*Data is shown as mean ± standard deviation; ^∗^at the end of the treatment period; weight and blood pressure analyzed by two-way ANOVA with Tukey’s post hoc analysis; age analyzed by one-way ANOVA with Tukey’s post hoc analysis, NTL, non-transgenic littermates.*

### Functional Magnetic Resonance Imaging

All MRI scans were performed on a 7/16 small animal MR Pharmascan (Bruker Biospin GmbH, Ettlingen, Germany) equipped with an actively shielded gradient set of 760 mT/m with a 80 μs rise time and operated by a Paravision 6.0 software platform (Bruker Biospin GmbH, Ettlingen, Germany). Mice were anesthetized with an initial dose of 4% isoflurane (Abbott, Cham, Switzerland) in oxygen/air (200/800 μl/min) mixture and anesthesia were maintained at 1.5% isoflurane in oxygen/air (100/400 μl/min) mixture. Mice were placed in prone position on a water-heated support to keep body temperature within 36.5 ± 0.5°C. Body temperature was monitored with a rectal temperature probe.

Perfusion MRI was performed using an arterial spin labeling technique as described previously (Ni et al., 2018a). The Paxinos mouse brain atlas was used as anatomical reference for scan positioning and analysis (Paxinos and Franklin, 2012). Re-assessment of CBF (test–retest analysis) was performed in 17 mice randomly selected from the groups for assuring the repeatability of the CBF measurement (*n* = 17).

Cerebral vascular reactivity was assessed, after a 1 week of recovery following perfusion MRI, by measuring cerebral blood volume (CBV) changes upon pharmacological stimulation using vessel dilator acetazolamide, as previously reported (Mueggler et al., 2002; Princz-Kranz et al., 2010). The scanner was equipped with a 300 MHz cryogenic radiofrequency probe for conducting the CVR assay. Mice were endotracheally-intubated and maintained at 1.5% isoflurane in oxygen/air (100:400 μl/min) mixture and actively ventilated at a rate of 90 breaths/minute and a tidal volume of approximately 0.3 μl/breath using a small animal ventilator (MRI-1, CWE, Inc., United States). Animal’s tail veins were cannulated for administration of drugs and contrast agent. A neuromuscular blocking agent gallanmine triethiode (Sigma-Aldrich GmbH, Switzerland) was administered twice as a bolus (40 μl, 7 mg/ml) at the beginning and before *i.v.* injection of contrast agent while the isoflurane level was reduced to 1.2%. T_2_-weighted anatomical reference images were acquired using a spin echo rapid acquisition with relaxation enhancement (RARE) sequence with the same anatomical geometry as RARE sequence at pre- and post-injection of vessel dilator acetazolamide. Fifteen minutes after injection of contrast agent, of gallanmine was injected *i.v.* (21 mg/kg body weight). Then, eight sequential pre-contrast agent scans at baseline signal intensity *S*_pre_ (CBV_0_ image) were acquired using a RARE sequence: temporal resolution = 40 s, repetition time = 3333 ms, echo time _eff_ = 81 ms, RARE factor = 32, field-of-view = 20 mm × 20 mm, imaging matrix = 133 × 103, slice thickness = 1 mm, 1.5 mm gap, resolution = 150 μm × 200 μm. FLASH sequence NR = 50 was used for ensuring the successful injection of the contrast agent. After 10 repetitions, iron oxide contrast agent Endorem (50 mg Fe/kg body weight, Guerbet SA, Roissy-en-France, France) was administered *i.v.* A RARE sequence was started after 5 min after repetition = 100 and 8 averages, when the contrast agent concentration had reached steady state. After the 30^th^ repetition (20 min), acetazolamide (30 mg/kg body weight; Diamox^®^ parenteral, Goldshield Pharmaceuticals, Ltd., Croydon, United Kingdom) was administered *i.v.* as a bolus. Additional 70 images were collected (46.7 min), yielding the image series *S*_(_*_t_*_)_.

### Brain Tissue Collection

After MRI, mice were anesthetized under ketamine/xylazine/acepromazine maleate (75/10/2 mg/kg body weight, bolus injection, *i.p.*). Mice were perfused using cold PBS (pH 7.4). Brains were removed from the skull. The left brain hemispheres were fixed in 4% paraformaldehyde (Sigma-Aldrich, Switzerland) in PBS (pH 7.4) at 4°C and embedded in paraffin following routine procedures. The right hemispheres were snap frozen and stored at -80°C, until use.

### Meso Scale Discovery (MSD) Analysis

Meso Scale Discovery (MSD) analysis was performed as described previously (Kulic et al., 2012) in NTL (*n* = 6), NTL noscapine (*n* = 8), arcAβ (*n* = 7), and arcAβ noscapine (*n* = 4) mice. In brief, frozen brain tissues were homogenized using a glass Teflon homogenizer with homogenization buffer containing 100 mM Tris-HCl, 150 mM NaCl and complete EDTA-free protease inhibitor cocktail (Roche Diagnostic, Risch-Rotkreuz, Switzerland). The amount of buffer used was equivalent to 10-fold wet weight of the brain tissue. The brain homogenates were centrifuged at 100,000 *g* for 1 h and supernatants were collected Tris-buffered saline (TBS) fraction and pellets were homogenized again in homogenization buffer containing 2% of sodium dodecyl sulfate (SDS). Centrifugation was repeated and the supernatants (SDS-fraction) were again collected. The remaining pellet was suspended in 70% formic acid (FA), sonicated four times for 30 s at 30% power and again centrifuge at 100,000 *g* for 30 min. The supernatants were collected (FA-fraction), lyophilized and reconstituted in homogenization buffer. Aβ_38_, Aβ_40_, and Aβ_42_ levels were measured in the abovementioned brain homogenate fractions using an MSD 3plex multi-SPOT Aβ human kit (Gaithersburg, MD, United States). Plates were measured on an MSD SECTOR Imager 600 plate reader (Gaithersburg, MD, United States). The MSD DISCOVERY WORKBENCH software (Version 3.0.18) (Gaithersburg, MD, United States) with Data Analysis Toolbox was used to calculate sample concentrations by comparing them against a standard curve.

### Immunohistochemistry

Cy3-6E10 (mouse anti-Aβ-SIG-39320, Signet Laboratories, United States, 1:5000 dilution) and Alexa488-ionized calcium-binding adapter molecule (Iba1, Rabbit anti-Iba1, Wako Chemicals, United States, HRP Rabbit AK, 1:2500 dilution) immunostainings were done on 5 μm brain sections as previously described (Spani et al., 2015). Nuclei were counterstained by DAPI, while adjacent sections were stained with hematoxylin and eosin. Images were examined using Leica DM 4000B microscopy with Olympus DP71 digital camera and VIS (Visiopharm Integrator System) version 4.4.6.9 software at 5×, 10×, 20×, and 40× magnification. Images were captured on each slices at (× 5, 10, 20, 40 magnification). Quantification of the images was performed blinded using Image J (NIH, United States). For quantitative image analysis, the cortex and hippocampus were analyzed on three equidistant sagittal hemibrain sections of each mouse (interaural lateral 0.60 to 0.72 mm) at 10×, three images taken at each region on one slice. Images of the cortex and hippocampus were taken with an exposure time of 200 ms (amyloid, 6E10-Cy3), 308 ms (microglia, Iba1-Alexa488), 30 ms (nucleus, DAPI) of the same areas. The Paxinos mouse brain atlas was used as anatomical reference (Paxinos and Franklin, 2012). Threshold and particle analysis functions were applied, resulting in Aβ and microglia covered area in percentage.

### Data Analysis

Two persons performed data analysis independently, and the obtained results were averaged. CBF maps from arterial spin labeling data was computed as previously described (Ni et al., 2018a). Region of interest (ROIs) were evaluated in the cerebral cortex, hippocampus and thalamus using AFNI (NIH, United States) with T_2_-weighted anatomical scan and the Paxino mouse brain atlas as reference (Paxinos and Franklin, 2012). Figure 2A shows the placement of the ROIs on the anatomical scans, which were transferred to the CBF map. CBF in ROIs was calculated from both T_1_ values (Zerbi et al., 2014) using MATLAB R2015b (The Mathworks, Natick, MA, United States):

(1) $$CBF/\lambda =\frac{T_{1}\;global}{T_{1}\;blood}(\frac{1}{T_{1}\;selective}-\frac{1}{T_{1}\;global})$$

where λ is the blood/tissue partition coefficient for water, assumed to be 0.9 μl/g (Leithner et al., 2008) and T_1_ blood was assumed to be 2.007 s at 7 T (Zhang et al., 2013).

Assessment of CVR was performed using Biomap 6 (Novartis Institute for Biomedical Research, Basel, Switzerland). The ROIs were defined in six brain regions (motor cortex, sensory cortex, striatum, hippocampus, thalamus, and cerebellum). Baseline CBV (CBV_0_) was calculated for each ROI. The baseline CBV and relative percentage changes of CBV versus baseline CBV (ΔCBV(*t*)) were computed on a pixel-by-pixel basis according to:

(2) $$\Delta CBV\%(t)=\frac{\Delta CBV(t)}{CBV_{0}}\times 100=\frac{\ln(S(t))/\ln(S_{0})}{\ln(S_{0})/\ln(S_{pre})}\times 100$$

(3) $${CBV}_{0}=-\ln(\frac{T}{T_{0}})=-\ln(\frac{mean\;Post\;CA}{mean\;PreCA})$$

(4) sqrt(ΔCBV%(t))=a×time+b

Linear regression analysis was performed on the square root of the Δ*CBV% (t)*, sqrt(Δ CBV% (t)) with time. ROIs of specific brain regions in the left and right hemispheres were merged into one ROI. Figure 3A shows the placement of the ROIs for analyzing the CBV data using T_2_-weighted anatomical scan and the Paxino mouse brain atlas as reference (Paxinos and Franklin, 2012). The response time curve ΔCBV (*t*) and the late ΔCBV(%) of slices 90–100 (40–46.7 min) were compared between groups.

### Statistical Analysis

For comparison of the continuous numeric data such as weight, blood pressure before and after treatment, a two-way ANOVA with Tukey’s *post hoc* analysis (GraphPad Prism 6.0, San Diego, CA, United States) was used. For comparison of age between groups, a one-way ANOVA with Tukey’s *post hoc* analysis was used. For comparison of bradykinin plasma level between NTLs and arcAβ mice, unpaired *t*-test was used after Shapiro–Wilk normality test. For group comparison of CBF, CBV_0_, late ΔCBV(%), slope of sqrt(ΔCBV%(t)) and immunostaining of Aβ and Iba1 in multiple brain regions, two-way ANOVA with Turkey’s *post hoc* analysis were used. For MSD measurement of Aβ level in different brain fractions of test–retest analysis of the CBF data of the same mice, a Pearson correlation analysis was performed. All data are present as mean ± standard deviation. Significance was set at ^∗^*p* < 0.05, ^∗∗^*p* < 0.01, ^∗∗∗^*p* < 0.001.

## Results

### Demographics and Blood Pressure

Demographic information, body weight before/after the treatment and blood pressure are summarized in Table 1 including NTL (*n* = 8), NTL noscapine (*n* = 9), arcAβ (*n* = 9), and arcAβ noscapine (*n* = 11). One-way ANOVA showed that groups did not differ in terms of age among the groups (approximately 16.6 months-old after the treatment), F(DFn, DFd): *F*(3,33) = 0.4467, *p* = 0.7212. And no differences between body weights before and after the treatment period were found between groups *F*(3,66) = 0.104, *p* = 0.9574. As inhibitors of BRs can affect the angiotensin system (AbdAlla et al., 2001; Jing et al., 2012), we measured the systolic and diastolic blood pressure in mice at the end of the treatment period. Blood pressure was not statistically different between noscapine-treated NTLs, and arcAβ mice compared to untreated groups *F*(3,33) = 0.1594, *p* = 0.9229 (Table 1).

### Increased Plasma Levels of Bradykinin 1–5 in arcAβ Mice

**FIGURE 1:**
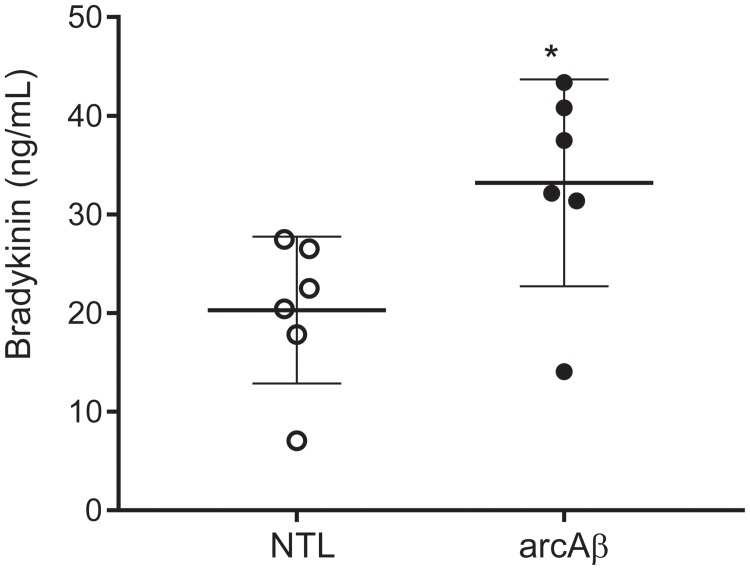
Bradykinin levels in the plasma of arcAβ mice and non-transgenic littermates (NTLs) measured by using enzyme immunoassay. ArcAβ mice (*n* = 6) have higher levels of circulating bradykinin, as compared to NTL (*n* = 6); ^∗^*p* < 0.05, unpaired Student’s *t*-test; Shapiro–Wilk *p* = 0.2514 and *p* = 0.3587, respectively passing normality test.

We tested with enzyme immunoassay if kallikrein-kinin system is activated in the arcAβ mouse model of amyloidosis. We detected that arcAβ mice have higher levels of circulating bradykinin in the plasma, as compared to NTLs (33.2 ± 10.5 vs. 20.3 ± 7.43 ng/ml, *p* = 0.0338, Shapiro–Wilk *p* = 0.2514 and *p* = 0.3587 respectively, passing normality test) (Figure 1).

### Noscapine Treatment Ameliorated Regional Hypoperfusion

**FIGURE 2:**
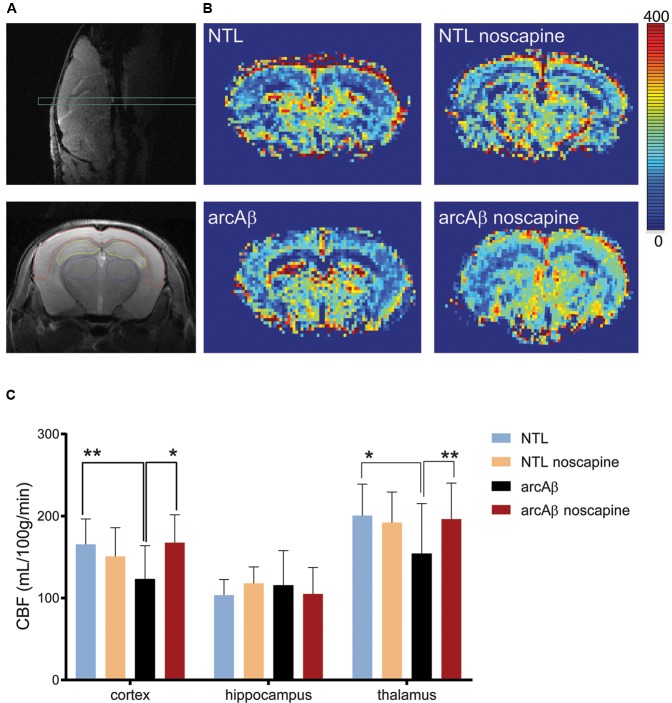
Bradykinin receptor blockade by noscapine alleviates regional hypoperfusion in arcAβ mice. **(A)** Anatomical position of slice for arterial spin labeling on sagittal T_2_-weighted image (top) and representative regions of interest (ROI) [cortex (red), hippocampus (yellow), and thalamus (blue)] on coronal T_2_-weighted anatomical image acquired at the same position (bottom). **(B)** Representative coronal CBF maps (approximately Bregma –1.5 ± 0.3 mm) of untreated and noscapine-NTLs and arcAβ mice, respectively. **(C)** Quantification of regional CBF showed hypoperfusion in the cortex and thalamus of arcAβ compared with NTLs that is mitigated by noscapine treatment. Data are present as mean ± standard deviation; ^∗^*p* < 0.05, ^∗∗^*p* < 0.01, two-way ANOVA with *post hoc* Turkey’s correction for multiple comparison; CBF, cerebral blood flow. NTL (*n* = 8), NTL noscapine (*n* = 9), arcAβ (*n* = 9), arcAβ noscapine (*n* = 11).

We first tested the effect of chronic oral noscapine treatment on cerebral perfusion of arcAβ mice. Figure 2A shows the placement of acquisition slice and ROIs of CBF analysis on the structural MRI image. Figure 2B shows representative coronal CBF maps for all four groups of mice. Regional analysis of the perfusion MRI data showed the interaction between brain region and treatment × genotype with F(DFn,DFd): *F*(6,186) = 2.316, *p* = 0.0351; reduced CBF in the cortex (123.0 ± 40.8 vs. 165.4 ± 31.1 μl/100 g/min, *p* = 0.0192) and thalamus (154.1 ± 61.1 vs. 200.5 ± 38.4 μl/100 g/min, *p* = 0.0065) in untreated arcAβ mice compared to untreated NTLs were observed (Figure 2C). Noscapine-treated arcAβ mice showed significantly higher CBF values in the cortex (167.6 ± 33.9 vs. 123.0 ± 40.8 μl/100 g/min, *p* = 0.0023) and thalamus (196.3 ± 43.8 vs. 154.1 ± 61.1 μl/100 g/min, *p* = 0.0043) compared to untreated arcAβ mice. The test–retest results of two perfusion MRI measurements in the same mice are shown in Supplementary Figure 2. Pearson correlation analysis showed a robust correlation (*r* = 0.9491, *p* < 0.0001, *n* = 17) between test and re-test measures. Taken together, perfusion imaging revealed that noscapine treatment mitigated the hypoperfusion seen in arcAβ mice of this age.

### Noscapine Treatment Mitigated Impaired CVR

**FIGURE 3:**
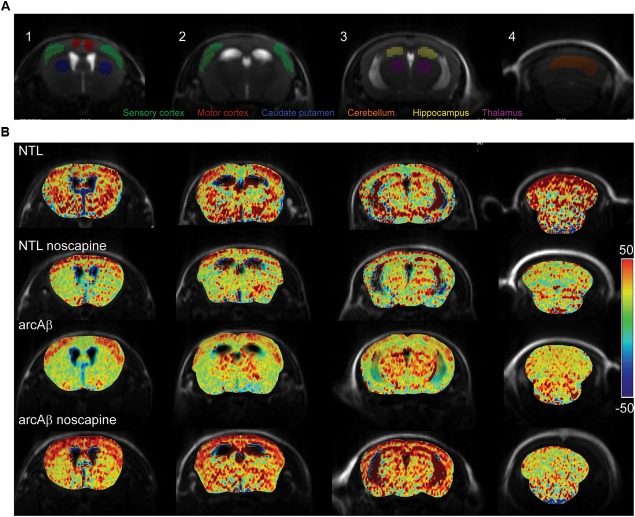
Bradykinin receptor blockade ameliorates the reduced regional cerebral vascular reactivity in noscapine-treated arcAβ mice. **(A)** Representative ROI on coronal anatomical T_2_-weighted MR images at showing the motor cortex, sensory cortex, hippocampus, thalamus, striatum, and cerebellum. **(B)** Representative maps of percentage change in cerebral blood volume (ΔCBV) of untreated and noscapine-treated NTLs and arcAβ mice. Data are present as mean ± standard deviation; ΔCBV: percentage change in CBV.

We then tested the effect of noscapine treatment on the CVR by measuring CBV after stimulation with vessel dilator acetazolamide. One mouse in the un-treated NTLs, noscapine-treated NTLs and noscapine-treated arcAβ group showed negative ΔCBV and were categorized as outliers and thus excluded from further analysis. Figure 3A illustrated the ROIs drawing on four different levels of MR slices identified using the Paxinos mouse brain atlas for CBV analysis (Paxinos and Franklin, 2012). Figure 3B illustrated the representative ΔCBV of different anatomical levels of mice at 46 min after-the injection of acetazolamide (*t* = 0 min) overlaid with corresponding T_2_-weighted MRI. No difference was observed in baseline CBV_0_ values among groups [two-way ANOVA with Tukey’s *post hoc* analysis, *F*(15,186) = 0.1919, Supplementary Figure 3].

**FIGURE 4:**
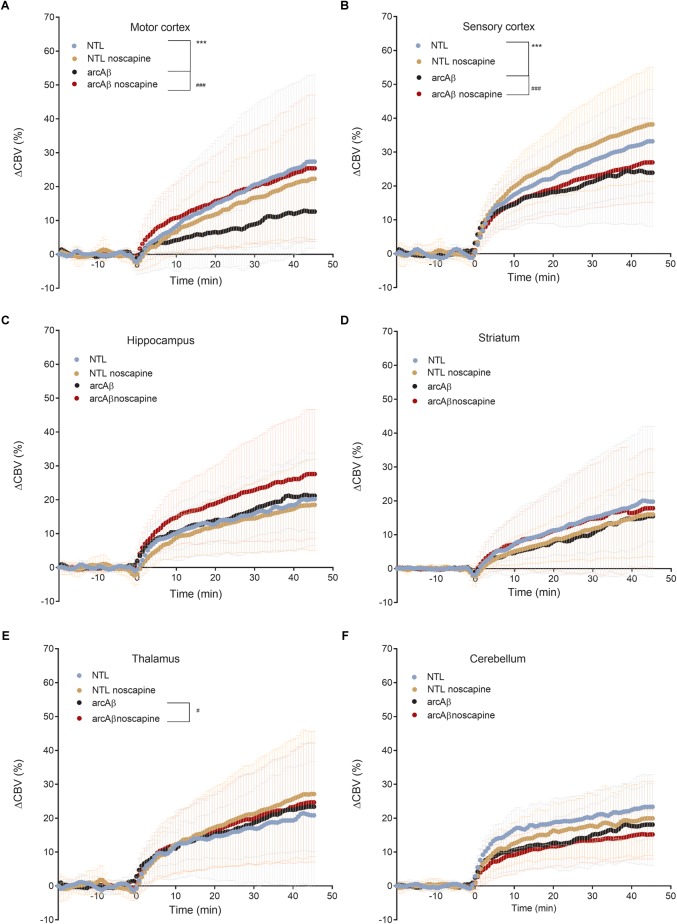
Regional cerebral vascular reactivity in mouse brain. **(A–F)** Percent change in cerebral blood volume (ΔCBV) plotted as a function of time for six brain regions (motor cortex, sensory cortex, hippocampus, striatum, thalamus, and cerebellum) of untreated and noscapine-treated NTLs and arcAβ mice, respectively. Injection of acetazolamide at *t* = 0 minute. Data are present as mean ± standard deviation; ^∗∗∗^*p* < 0.001 NTL vs. arcAβ, ^#^*p* < 0.05, ^##^*p* < 0.01, and ^###^*p* < 0.001 arcAβ vs. arcAβ-noscapine, two-way ANOVA with *post hoc* Tukey’s correction for multiple comparison. NTL (*n* = 8), NTL noscapine (*n* = 9), arcAβ (*n* = 9), and arcAβ noscapine (*n* = 11).

In Figure 4 the change in ΔCBV is plotted as a function of time for the four groups of mice (*t* = -20 min: contrast agent injection; *t* = 0 min: acetazolamide stimulus). Linear regression analysis were performed on sqrt (%ΔCBV) with time (0–46.7 min). Two way ANOVA with Tukey’s *post hoc* analysis showed significant interaction *F*(15,222) = 11.92, *p* < 0.0001. The slope and intercept and goodness of the fitting are summarized in Table 2. A flatter slope of sqrt(%ΔCBV(t)) was observed in the motor cortex (*p* < 0.001), sensory cortex (*p* < 0.001) of untreated arcAβ compared to untreated NTLs. A significantly steeper slope was observed for noscapine-treated arcAβ compared to untreated arcAβ mice in the motor cortex (*p* = 0.0096), sensory cortex (*p* < 0.0001), and thalamus (*p* = 0.0161).

**Table 2:** Linear regression analysis of square root (ΔCBV%) along with time in mice.

Region		NTL	NTL noscapine	arcAβ	arcAβ noscapine
Motor cortex	Slope	0.061 ± 0.002	0.066 ± 0.002	0.048 ± 0.001^∗∗∗^	0.057 ± 0.001^##^
	Intercept	2.178 ± 0.056	1.747 ± 0.047	1.4 ± 0.023	2.379 ± 0.037
	*R*	0.9243	0.9523	0.9773	0.9614
Sensory cortex	Slope	0.064 ± 0.003	0.069 ± 0.004	0.038 ± 0.002^∗∗∗^	0.054 ± 0.003^###^
	Intercept	3.120 ± 0.087	3.383 ± 0.096	3.164 ± 0.058	2.989 ± 0.082
	*R*	0.8462	0.8399	0.8172	0.8162
Hippocampus	Slope	0.045 ± 0.002	0.047 ± 0.002	0.050 ± 0.002	0.050 ± 0.002
	Intercept	2.451 ± 0.047	2.127 ± 0.055	2.503 ± 0.048	2.959 ± 0.066
	*R*	0.9036	0.8816	0.917	0.8556
Thalamus	Slope	0.042 ± 0.002	0.078 ± 0.003	0.049 ± 0.002	0.058 ± 0.002^#^
	Intercept	2.549 ± 0.063	2.669 ± 0.090	2.690 ± 0.044	2.731 ± 0.062
	*R*	0.8179	0.8829	0.9258	0.8999
Caudate nucleus	Slope	0.054 ± 0.001	0.065 ± 0.001	0.059 ± 0.002	0.053 ± 0.002
	Intercept	1.826 ± 0.032	1.327 ± 0.034	1.376 ± 0.043	1.848 ± 0.045
	*R*	0.9666	0.9744	0.9515	0.9334
Cerebellum	Slope	0.035 ± 0.003	0.045 ± 0.003	0.035 ± 0.001	0.037 ± 0.002
	Intercept	3.342 ± 0.075	2.946 ± 0.075	2.619 ± 0.033	2.429 ± 0.050
	*R*	0.6884	0.7877	0.9198	0.8486

*Data is shown as mean ± standard error of the mean; ^∗∗∗^p < 0.001 NTL vs. arcAβ, ^#^p < 0.05, ^##^p < 0.01, and ^###^p < 0.001 arcAβ vs. arcA β-noscapine, two-way ANOVA with Tukey’s post hoc analysis; NTL (n = 8), NTL noscapine (n = 9), arcAβ (n = 9), arcAβ noscapine (n = 11).*

Two-way ANOVA with Tukey’s *post hoc* analysis showed the interaction between brain region and treatment × genotype with *F*(15,2244) = 8.3167, *p* < 0.0001; significantly reduced late ΔCBV (average of *t* = 40–46.7 min) was observed in untreated arcAβ mice compared to untreated NTLs in the motor cortex (12.5 ± 8.3 vs. 28.9 ± 26.1%, *p* < 0.0001), sensory cortex (24.0 ± 15.1 vs. 30.4 ± 11.8%, *p* = 0.0219) (Supplementary Figure 4). Higher late ΔCBV was observed in noscapine-treated arcAβ compared to untreated arcAβ mice in the motor cortex (24.6 ± 19.9 vs. 12.5 ± 8.3%, *p* < 0.0001) and hippocampus (26.9 ± 17.9 vs. 21.1 ± 10.0%, *p* = 0.0174). Taken together, measurements of CBV upon acetazolamide stimulation showed that noscapine treatment rescued the reduced CVR in cortical and subcortical regions of arcAβ mice.

### Noscapine Did Not Affect Microglia Activation and Beta-Amyloid Level

**FIGURE 5:**
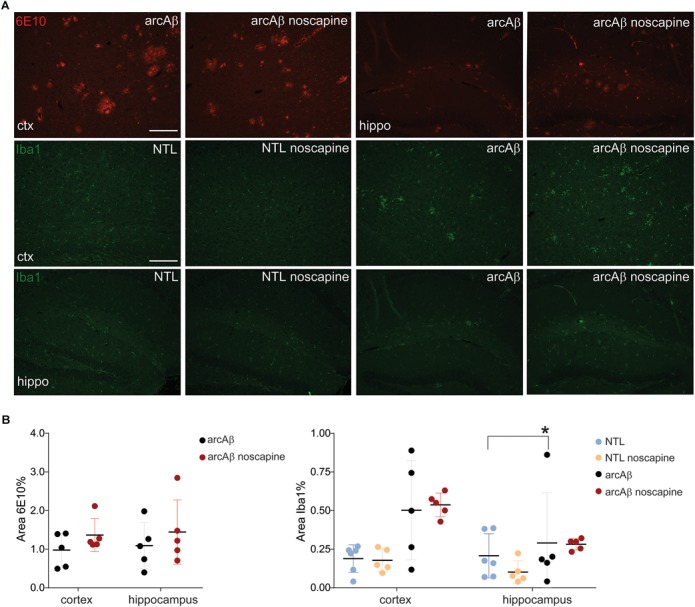
Immunohistochemical analysis of Aβ load and microglia activation. **(A)** Brain tissue sections of untreated and noscapine-treated NTLs and arcAβ mice were stained for amyloid (6E10, red), microglia (Iba1, green) immunoreactivity in the cortex (ctx) and hippocampus (hippo) of untreated and noscapine-treated NTLs and arcAβ mice, respectively. Scale bar = 400 μm. **(B)** Quantification of immunoreactivity. Data are present as mean ± standard deviation; Iba1: ionized calcium-binding adapter molecule 1. ^∗^*p* < 0.05, two-way ANOVA with Tukey’s *post hoc* correction for multiple comparison. NTL (*n* = 5), NTL noscapine (*n* = 5), arcAβ (*n* = 5), and arcAβ noscapine (*n* = 5).

Previous reports about the effect of B_1_R and B_2_R blockade on neuroinflammation and Aβ load in animal models of AD have been contradictory. We therefore analyzed microgliosis and Aβ load in all groups of mice by immunohistochemistry (Figure 5). Two-way ANOVA analysis indicated that noscapine treatment had no influence on amyloid burden in the arcAβ mice as revealed by quantitative analysis of 6E10 immunostainings in the cortex (% 6E10 area, 1.37 ± 0.42 vs. 0.97 ± 0.44, ns) or in the hippocampus (1.44 ± 0.83 vs. 1.09 ± 0.59, ns), Tukey’s *post hoc* analysis *F*(1,16) = 0.004815, *p* = 0.9455). Moreover, microgliosis Iba1 immunoreactivity was significantly higher in the cortex (0.50 ± 0.32 vs. 0.19 ± 0.09, *p* = 0.0279) of untreated arcAβ mice compared to NTLs, but not in the hippocampus. The microgliosis Iba1 immunoreactivity was similar between noscapine-treated and untreated NTLs in the cortex (0.18 ± 0.07 vs. 0.19 ± 0.09, ns), and the hippocampus (0.10 ± 0.07 vs. 0.21 ± 0.14, ns); and between noscapine-treated and untreated arcAβ mice in the cortex (0.53 ± 0.07 vs. 0.50 ± 0.32, ns) and the hippocampus (0.28 ± 0.04 vs. 0.21 ± 0.14, ns) [two-way ANOVA with Tukey’s *post hoc* analysis *F*(3,34) = 1.399, *p* = 0.2599]. These results suggest that B_1_R and B_2_R blockade with noscapine at this dosage and duration did not alter the amyloid load and degree of Aβ-associated microgliosis.

#### MSD Analysis

**FIGURE 6:**
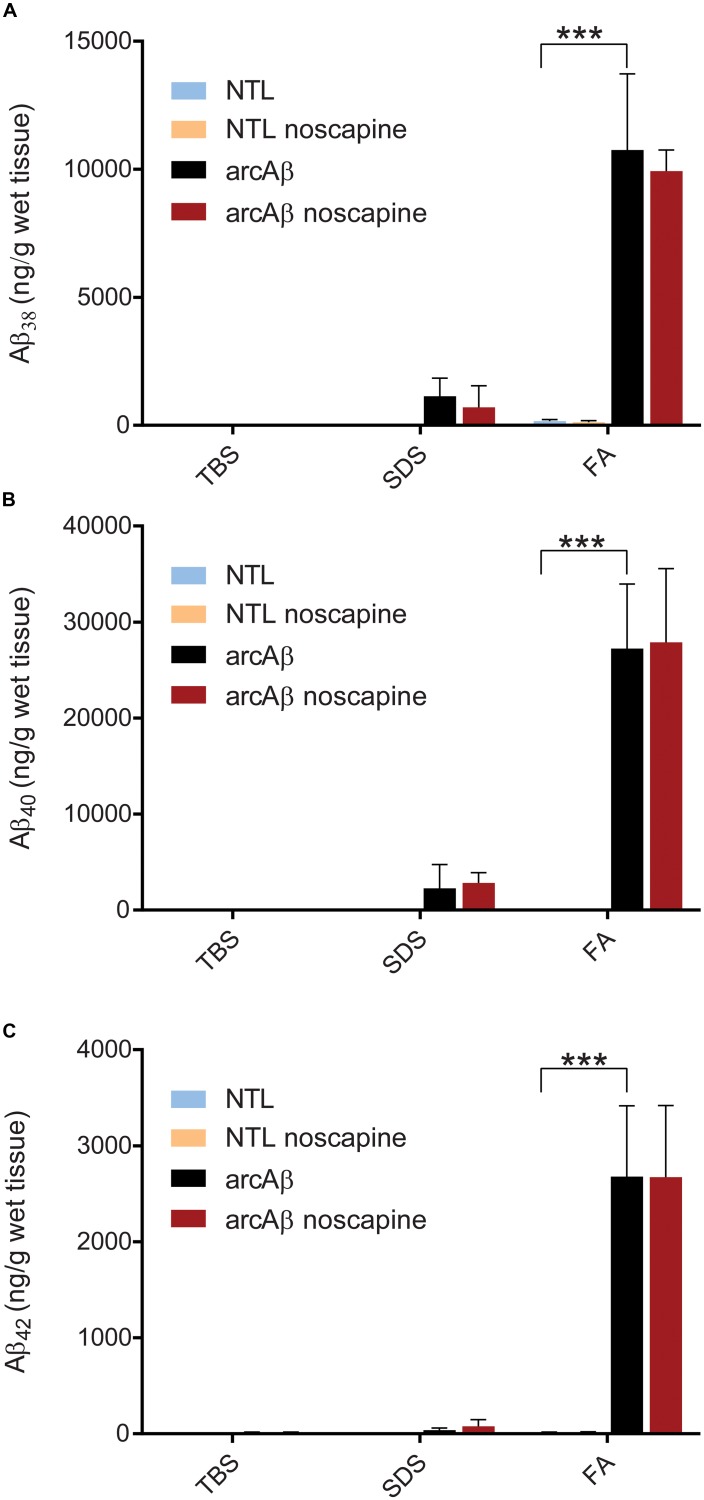
Meso Scale Discovery (MSD) analysis of Aβ load in TBS, SDS and FA fraction of mouse brain homogenates from untreated and noscapine-treated NTLs and arcAβ mice **(A)** Aβ_38_; **(B)** Aβ_40_; **(C)** Aβ_42_. ^∗∗∗^*p* < 0.001, two-way ANOVA with Tukey’s *post hoc* correction for multiple comparison. NTL (*n* = 6), NTL noscapine (*n* = 8), arcAβ (*n* = 7), and arcAβ noscapine (*n* = 4) mice.

We used MSD immunoassay to determine Aβ levels in three protein fractions from the brain hemispheres. Two-way ANOVA with Tukey’s *post hoc* analysis showed greater amount of Aβ_38_ (10747.1 ± 2977.4 vs. 164.8 ± 57.8, *p* < 0.0001), Aβ_40_ (27222.3 ± 6376.1 vs. 35.5 ± 9.0, *p* < 0.0001), and Aβ_42_ (2679.3 ± 737.7 vs. 14.3 ± 4.7, *p* < 0.0001) in FA fractions brain homogenates (Figure 6) from arcAβ mice (*n* = 7) compared to NTLs (*n* = 6). No significant difference was detected in the level of Aβ_38_, Aβ_40_ and Aβ_42_ in TBS, SDS, and FA fractions between noscapine-treated arcAβ mice (*n* = 4) and untreated arcAβ mice (*n* = 7).

## Discussion

In the current study, we showed using fMRI techniques a pronounced vascular dysfunction in the arcAβ mouse model of cerebral amyloidosis. Chronic administration of B_1_R and B_2_R antagonist noscapine ameliorated amyloidosis-associated cerebral hypoperfusion and vascular reactivity, but did not affect Aβ cerebral load and associated microgliosis.

### Activation of the Kallikrein-Kinin System in a Transgenic Mouse Model of Cerebral Amyloidosis

The kallikrein-kinin system has been shown to be activated both in patients with AD and in animal disease models (Bergamaschini et al., 1998; Viel and Buck, 2011; Lacoste et al., 2013). In the central nervous system, BRs are mainly distributed in the cortex and hippocampus, the brain structures that are affected earliest by AD pathologies (Jong et al., 2002; Ashby et al., 2012). In the brain B_1_R and B_2_R are expressed in a variety of cells including neurons, astrocytes, microglia, oligodendrocytes, and endothelial cells. Upregulation of brain BR levels was shown in rats (Viel et al., 2008), mice (Prediger et al., 2008) in response to infusion of human Aβ, as well as in transgenic mouse models of cerebral amyloidosis (Lacoste et al., 2013; Passos et al., 2013). Also, increased B_2_R density was demonstrated by ^3^H-bradykinin binding assay in skin fibroblasts from patients with AD compared to that from age-matched healthy controls (Noda et al., 2003). In the present study, we showed that arcAβ mice of amyloidosis have increased plasma bradykinin levels as compared to NTLs at the endpoint of the study. The arcAβ mouse model of cerebral amyloidosis exhibits cerebral plaque deposition from 6 months-of-age together with strong congophilic cerebral amyloid angiopathy (Knobloch et al., 2007) and increased bradykinin plasma levels may likely be due to vascular amyloid deposition.

### BRs as Pharmacological Targets in AD

Previous studies using animal models of AD have described BRs blockade as a potential treatment strategy for AD. Pharmacological blockade of both BRs in transgenic mice over-expressing amyloid precursor protein or mice infused with Aβ has been shown to improve learning and memory formation. B_1_R blockade mitigated cognitive deficits by B_1_R antagonist des-Arg9[Leu8]-bradykinin in Aβ-treated mice (Prediger et al., 2008) and in rat (Bitencourt et al., 2017), and by B_1_R antagonist SSR240612 in Tg-SwDI mice of AD (Lacoste et al., 2013). B_2_R blockade by HOE 140 protected against cognitive impartment in Aβ-infused mice (Prediger et al., 2008; Bicca et al., 2015).

It has been described that B_2_Rs are constitutively expressed and mediate most physiological actions of kinins, whereas B_1_Rs are highly inducible upon inflammatory stimulation and tissue injury (Marceau et al., 2002). Thus, the majority of previous preclinical studies have focused on B_1_R inhibition. However, it has been described that the expression of both receptors is increased under pathological conditions related to oxidative stress, pro-inflammatory stimuli (e.g., inflammation and infection) as well as vasoactive peptides of the renin-angiotensin system (Nokkari et al., 2018). Thus, we have used noscapine a phthalideisoquinoline alkaloid, which is an antagonist for both B_1_R and B_2_R (pA2 = 6.68) (Mahmoudian and Mojaverian, 2001). We decided to provide the drug in drinking water as an easy administrable way for chronic pharmacological treatment, but which might have introduced variability in the treatment effect due to different uptake of the drug by the mice. Noscapine has been clinically approved as antitussive drug, and has the advantage that it can be administered orally, that its pharmacokinetics and metabolic fate are characterized, and that it has low toxicity (Empey et al., 1979; Tsunoda and Yoshimura, 1979; Karlsson et al., 1990).

### Blockade of B_1_R and B_2_R Ameliorates Cerebrovascular Dysfunction

We used established functional MRI protocols to assess the effects of noscapine treatment on cerebrovascular dysfunction *in vivo* (Klohs et al., 2014; Ni et al., 2018a). Cerebrovascular dysfunction is an early pathological event in patients with AD (Iturria-Medina et al., 2016). The brain critically depends on the continuous supply of oxygen and nutrients for proper cognitive function, and thus CBF is a tightly regulated process. In a previous study it was shown with Laser Doppler flowmetry in the J20 mouse model that blockade of B_1_R improves CBF (Lacoste et al., 2013). We have assessed CBF using a non-invasive arterial spin labeling technique. We showed a 25% reduction in CBF in the cortex of approximately 17-months-old arcAβ mice compared to age-matched NTLs. In a previous study using the same fMRI technique we found normal CBV values in 6-months-old arcAβ mice and a 30% reduction in CBF in the cortex of 24-months-old arcAβ mice compared to NTLs (Ni et al., 2018b), which indicates that the severity of the CBF reduction increased with increasing age in that mouse model. With perfusion MRI, we observed that noscapine treatment reversed the pathological hypoperfusion in the cortex and thalamus in arcAβ mice.

Cerebral vascular reactivity reflects the capacity of blood vessels to dilate and is an important indicator of the brain vascular reserve capacity. Dynamic measurement of CBV upon acetazolamide challenge is commonly used in humans and animals for quantification of CVR and has shown to be impaired in AD patients and models of cerebral amyloidosis (Stoppe et al., 1995; Mueggler et al., 2002; Princz-Kranz et al., 2010; Dumas et al., 2012). Acetazolamide inhibits carbonic anhydrase, which results into increased CBV, but its exact mode of action is not yet clear (Vorstrup et al., 1984; Dumas et al., 2012). In our study we observed a global reduction in CVR in the cortical and subcortical regions in untreated arcAβ mice compared to NTLs. A previous fMRI study has described a reduction of CVR in cortical regions but not subcortical regions in 16-months-old arcAβ mice that was aggravated with aging (Princz-Kranz et al., 2010). These differences in CVR in subcortical regions of arcAβ mice might be due to the fact that mice although being of the same strain were from different colonies. Biological factors such as genetic backgrounds (our arcAβ mice were derived from transgenic mouse strain back-crossed to C57BL6 for over 10 generations compared to mice in the previous study which had been on a mixed genetic background) and environmental factors such as housing conditions can affect the phenotype. In the current study we found that treatment with noscapine ameliorated CVR reduction in arcAβ mice, which is in line with a previous study that blockade of B_1_R significantly ameliorated sensory-evoked hemodynamic responses in the J20 mouse model (Lacoste et al., 2013). As inhibition of BRs can affect the angiotensin system (AbdAlla et al., 2001; Jing et al., 2012), we measured systolic and diastolic blood pressure between groups. As we did not observe differences in blood pressure values among groups, we ruled out that the observed ΔCBV differences were due to actions of noscapine on the angiotensin system.

The findings from CVR and CBF measurement clearly demonstrate that inhibition of B_1_R and B_2_R ameliorate Aβ-induced cerebrovascular dysfunction. The molecular mechanisms underlying cerebrovascular dysfunction in AD are not well-understood and it can only be speculated how noscapine treatment ameliorated hypoperfusion and reduced CVR in arcAβ mice. It has been suggested that blockade of BRs antagonized the production of vascular reactive oxygen species and inflammatory cytokines (Lacoste et al., 2013; Nokkari et al., 2018), which might have contributed to the restoration of the vascular reactivity (Klohs et al., 2012).

### Blockade of B_1_R and B_2_R Does Not Alter Neuropathology

In addition to functional vascular effects of noscapine treatment, we also assessed the effects on neuroinflammation and Aβ pathology. Gliosis is an early phenomenon both in mouse models of amyloidosis (Rodriguez-Vieitez et al., 2015; Lopez-Picon et al., 2018) and in patients with AD (Heneka et al., 2015). Activated microglia surrounding amyloid deposits and upregulated pro-inflammatory cytokines have been reported in the arcAβ mice (Knobloch et al., 2007; Klohs et al., 2012) and we have seen also microglia activation in the brains of noscapine-treated and untreated arcAβ mice.

B_1_R is involved in inflammation in the central nervous system mainly through the activation of microglia (Asraf et al., 2017) and astrocytes (Lacoste et al., 2013). However, the effects of bradykinin inhibition on microglia and astrocytes have been inconsistent, both suppression (Bitencourt et al., 2017) and stimulation (Asraf et al., 2016) of neuroinflammation has been reported, or showed no effect (Asraf et al., 2016). Using measurements of Iba1 immunoreactivity in few selected animals from each group, we found no effect of chronic oral noscapine treatment on microglia activation. In the current study, we have used Iba1 immunoreactivity as a rough estimate of neuroinflammation and have not attempted to discriminate the different types of microglia (resting and activated), which would require a more elaborate histological analysis. In addition, other markers of neuroinflammation (for example GFAP, CD68, etc.) and larger groups of animals might be investigated.

Reports on the effect of B_1_R and B_2_R on Aβ load have also been contradictory. B_2_R activation promoted α-secretase processing of amyloid precursor protein in skin fibroblasts from patients with AD, resulting in a lower production of Aβ (Nitsch et al., 1998). B_1_R antagonist SSR240612 reduced Aβ deposition (Lacoste et al., 2013) in Tg-SwDI mouse model of AD amyloidosis, while another B_1_R antagonist R-715 treatment increased Aβ_40_ and fibrillar Aβ deposition in both transgenic mouse models of AD, Tg-SwDI (Passos et al., 2013) and 5 × familial mice (Asraf et al., 2016). Aβ can in turn activate the release of kinins in cultured endothelial cells (Joseph et al., 1999). In our study, using immunohistochemical analysis of brain section and MSD immunoassay of brain tissue we found no effect of inhibition of B_1_R and B_2_R inhibition on cerebral Aβ load.

### Methodological Considerations

The reported differences in the action of bradykinin receptor inhibitors might be due to the affinity of individual inhibitors to B_1_R and B_2_R, as well as other factors like pharmacokinetics, biodistribution of the drug used, and the timing, dose and duration of the treatment. In our study, we found amelioration of cerebrovascular dysfunction with chronic oral noscapine treatment, while the treatment seemed to have no effects on cerebral Aβ load and microglia activation. In that context, fMRI could constitute a more sensitive and earlier indicator of treatment response than histological or biochemical assessment. It might be, however, conceivable that a different dose of noscapine or longer treatment periods might have also led to detectable changes in neuropathology. In addition, we started treatment in arcAβ mice at an advanced disease stage, which limited the potential effect scale on the different read-outs. Importantly, we have not used behavioral read-outs to assess effects of noscapine treatment on the cognitive deficit in arcAβ mice (Knobloch et al., 2007) and thus do not know if noscapine has led to an improvement of cognitive function. Thus, more extensive studies using additional behavioral read-outs, and starting treatment at an early disease stage are needed to demonstrate the full potential of noscapine treatment on AD pathology.

## Conclusion

Long-term oral noscapine treatment alleviates brain hypoperfusion and restores CVR in an animal model of amyloidosis as revealed by fMRI. Blocking BRs represents a potential target for treatment of AD-associated cerebrovascular dysfunction.

## Availability of Data and Material

The datasets generated and/or analyzed during the current study are available in the repository http://doi.org/10.5281/zenodo.1209065.

## Author Contributions

RuN, LK, and JK conceived and designed the study and interpreted the results. RuN, DK, LL, and RW performed the experiments. RuN, RW, LL, LK, and JK analyzed the data. RuN and JK wrote the paper. All coauthors contributed constructively to the manuscript.

## Conflict of Interest Statement

The authors declare that the research was conducted in the absence of any commercial or financial relationships that could be construed as a potential conflict of interest.

## References

[B1] AbdAlla S. Lother H. El Massiery A. Quitterer U. (2001). Increased AT1 receptor heterodimers in preeclampsia mediate enhanced angiotensin II responsiveness. *Nat. Med.* 7:1003. 10.1038/nm0901-1003 11533702

[B2] Ashby E. L. Love S. Kehoe P. G. (2012). Assessment of activation of the plasma kallikrein-kinin system in frontal and temporal cortex in Alzheimer’s disease and vascular dementia. *Neurobiol. Aging* 33 1345–1355. 10.1016/j.neurobiolaging.2010.09.024 21074291

[B3] Asraf K. Torika N. Danon A. Fleisher-Berkovich S. (2017). Involvement of the Bradykinin B1 Receptor in microglial activation: in vitro and in vivo studies. *Front. Endocrinol.* 8:82. 10.3389/fendo.2017.00082 28469598 PMC5396024

[B4] Asraf K. Torika N. Roasso E. Fleisher-Berkovich S. (2016). Differential effect of intranasally administrated kinin B1 and B2 receptor antagonists in Alzheimer’s disease mice. *Biol. Chem.* 397 345–351. 10.1515/hsz-2015-0219 26556847

[B5] Bergamaschini L. Parnetti L. Pareyson D. Canziani S. Cugno M. Agostoni A. (1998). Activation of the contact system in cerebrospinal fluid of patients with Alzheimer disease. *Alzheimer Dis. Assoc. Disord.* 12 102–108. 10.1097/00002093-199806000-00008 9651139

[B6] Bicca M. A. Costa R. Loch-Neckel G. Figueiredo C. P. Medeiros R. Calixto J. B. (2015). B(2) receptor blockage prevents Aβ-induced cognitive impairment by neuroinflammation inhibition. *Behav. Brain Res.* 278 482–491. 10.1016/j.bbr.2014.10.040 25446751

[B7] Bitencourt R. M. Guerra De Souza A. C. Bicca M. A. Pamplona F. A. De Mello N. Passos G. F. (2017). Blockade of hippocampal bradykinin B1 receptors improves spatial learning and memory deficits in middle-aged rats. *Behav. Brain Res.* 316 74–81. 10.1016/j.bbr.2016.08.041 27566183

[B8] Braak H. Braak E. (1991). Neuropathological stageing of Alzheimer-related changes. *Acta Neuropathol.* 82 239–259. 10.1007/BF00308809 1759558

[B9] Caetano A. L. Dong-Creste K. E. Amaral F. A. Monteiro-Silva K. C. Pesquero J. B. Araujo M. S. (2015). Kinin B2 receptor can play a neuroprotective role in Alzheimer’s disease. *Neuropeptides* 53 51–62. 10.1016/j.npep.2015.09.001 26387425

[B10] Couture R. Lindsey C. Quirion R. Bjorklund A. Hokfelt T. (2000). Brain kallikrein- kinin system: from receptors to neuronal pathways and physiological functions. *Handb. Chem. Neuroanat.* 16 241–300. 10.1016/S0924-8196(00)80009-3

[B11] de la Torre J. C. (2004). Is Alzheimer’s disease a neurodegenerative or a vascular disorder? Data, dogma, and dialectics. *Lancet Neurol.* 3 184–190. 10.1016/S1474-4422(04)00683-0 14980533

[B12] Dumas A. Dierksen G. A. Gurol M. E. Halpin A. Martinez-Ramirez S. Schwab K. (2012). Functional MRI detection of vascular reactivity in cerebral amyloid angiopathy. *Ann. Neurol.* 72 76–81. 10.1002/ana.23566 22829269 PMC3408630

[B13] Empey D. W. Laitinen L. A. Young G. A. Bye C. E. Hughes D. T. (1979). Comparison of the antitussive effects of codeine phosphate 20 mg, dextromethorphan 30 mg and noscapine 30 mg using citric acid-induced cough in normal subjects. *Eur. J. Clin. Pharmacol.* 16 393–397. 10.1007/BF00568199 527635

[B14] Frisoni G. B. Fox N. C. Jack C. R. Jr. Scheltens P. Thompson P. M. (2010). The clinical use of structural MRI in Alzheimer disease. *Nat. Rev. Neurol.* 6 67–77. 10.1038/nrneurol.2009.215 20139996 PMC2938772

[B15] Heneka M. T. Carson M. J. El Khoury J. Landreth G. E. Brosseron F. Feinstein D. L. (2015). Neuroinflammation in Alzheimer’s disease. *Lancet Neurol.* 14 388–405. 10.1016/S1474-4422(15)70016-5 25792098 PMC5909703

[B16] Iturria-Medina Y. Sotero R. C. Toussaint P. J. Mateos-Perez J. M. Evans A. C. (2016). Early role of vascular dysregulation on late-onset Alzheimer’s disease based on multifactorial data-driven analysis. *Nat. Commun.* 7:11934. 10.1038/ncomms11934 27327500 PMC4919512

[B17] Jing F. Mogi M. Sakata A. Iwanami J. Tsukuda K. Ohshima K. (2012). Direct stimulation of angiotensin II type 2 receptor enhances spatial memory. *J. Cereb. Blood Flow Metab.* 32 248–255. 10.1038/jcbfm.2011.133 21971355 PMC3272601

[B18] Jong Y. Dalemar L. Seehra K. Baenziger N. (2002). Bradykinin receptor modulation in cellular models of aging and Alzheimer’s disease. *Int. Immunopharmacol.* 2 1833–1840. 10.1016/S1567-5769(02)00168-6 12489797

[B19] Joseph K. Shibayama Y. Nakazawa Y. Peerschke E. Ghebrehiwet B. Kaplan A. (1999). Interaction of factor XII and high molecular weight kininogen with cytokeratin 1 and gC1qR of vascular endothelial cells and with aggregated Abeta protein of Alzheimer’s disease. *Immunopharmacology* 42 203–210. 10.1016/S0162-3109(99)00136-8 10596854

[B20] Karlsson M. O. Dahlstrom B. Eckernas S. A. Johansson M. Alm A. T. (1990). Pharmacokinetics of oral noscapine. *Eur. J. Clin. Pharmacol.* 39 275–279. 10.1007/BF00315110 2257866

[B21] Kisler K. Nelson A. R. Montagne A. Zlokovic B. V. (2017). Cerebral blood flow regulation and neurovascular dysfunction in Alzheimer disease. *Nat. Rev. Neurosci.* 18 419–434. 10.1038/nrn.2017.48 28515434 PMC5759779

[B22] Klohs J. Baltes C. Princz-Kranz F. Ratering D. Nitsch R. M. Knuesel I. (2012). Contrast-enhanced magnetic resonance microangiography reveals remodeling of the cerebral microvasculature in transgenic ArcAbeta mice. *J. Neurosci.* 32 1705–1713. 10.1523/JNEUROSCI.5626-11.2012 22302811 PMC6703349

[B23] Klohs J. Deistung A. Ielacqua G. Seuwen A. Kindler D. Schweser F. (2016). Quantitative assessment of microvasculopathy in arcAβ mice with USPIO-enhanced gradient echo MRI. *J. Cereb. Blood Flow Metab.* 36 1614–1624. 10.1177/0271678X15621500 26661253 PMC5010097

[B24] Klohs J. Rudin M. Shimshek D. R. Beckmann N. (2014). Imaging of cerebrovascular pathology in animal models of Alzheimer’s disease. *Front. Aging Neurosci.* 6:32 10.3389/fnagi.2014.00032 PMC3952109 24659966

[B25] Knobloch M. Konietzko U. Krebs D. Nitsch R. (2007). Intracellular Abeta and cognitive deficits precede beta-amyloid deposition in transgenic arcAbeta mice. *Neurobiol. Aging* 28 1297–1306. 10.1016/j.neurobiolaging.2006.06.019 16876915

[B26] Kulic L. Mcafoose J. Welt T. Tackenberg C. Spani C. Wirth F. (2012). Early accumulation of intracellular fibrillar oligomers and late congophilic amyloid angiopathy in mice expressing the Osaka intra-Abeta APP mutation. *Transl. Psychiatry* 2:e183. 10.1038/tp.2012.109 23149447 PMC3565767

[B27] Lacoste B. Tong X. K. Lahjouji K. Couture R. Hamel E. (2013). Cognitive and cerebrovascular improvements following kinin B1 receptor blockade in Alzheimer’s disease mice. *J. Neuroinflammation* 10:57. 10.1186/1742-2094-10-57 23642031 PMC3710240

[B28] Landen J. W. Hau V. Wang M. Davis T. Ciliax B. Wainer B. H. (2004). Noscapine crosses the blood-brain barrier and inhibits glioblastoma growth. *Clin. Cancer Res.* 10 5187–5201. 10.1158/1078-0432.CCR-04-0360 15297423

[B29] Leithner C. Gertz K. Schrock H. Priller J. Prass K. Steinbrink J. (2008). A flow sensitive alternating inversion recovery (FAIR)-MRI protocol to measure hemispheric cerebral blood flow in a mouse stroke model. *Exp. Neurol.* 210 118–127. 10.1016/j.expneurol.2007.10.003 18037417

[B30] Lopez-Picon F. R. Snellman A. Eskola O. Helin S. Solin O. Haaparanta-Solin M. (2018). Neuroinflammation appears early on PET imaging and then plateaus in a mouse model of Alzheimer Disease. *J. Nuclic Med.* 59 509–515. 10.2967/jnumed.117.197608 28986511

[B31] Mahmoudian M. Mojaverian N. (2001). Efffect of noscapine, the antitussive opioid alkaloid, on bradykinin-induced smooth muscle contraction in the isolated ileum of the guinea-pig. *Acta Physiol. Hung.* 88 231–237. 10.1556/APhysiol.88.2001.3-4.5 12162581

[B32] Maier F. C. Wehrl H. F. Schmid A. M. Mannheim J. G. Wiehr S. Lerdkrai C. (2014). Longitudinal PET-MRI reveals beta-amyloid deposition and rCBF dynamics and connects vascular amyloidosis to quantitative loss of perfusion. *Nat. Med.* 20 1485–1492. 10.1038/nm.3734 25384087

[B33] Marceau F. Sabourin T. Houle S. Fortin J. Petitclerc E. Molinaro G. (2002). Kinin receptors: functional aspects. *Int. Immunopharmacol.* 2 1729–1739. 10.1016/S1567-5769(02)00189-3 12489786

[B34] Merlini M. Meyer E. P. Ulmann-Schuler A. Nitsch R. M. (2011). Vascular beta-amyloid and early astrocyte alterations impair cerebrovascular function and cerebral metabolism in transgenic arcAbeta mice. *Acta Neuropathol.* 122 293–311. 10.1007/s00401-011-0834-y 21688176 PMC3168476

[B35] Montagne A. Zhao Z. Zlokovic B. (2017). Alzheimer’s disease: a matter of blood-brain barrier dysfunction? *J. Exp. Med.* 214 3151–3169. 10.1084/jem.20171406 29061693 PMC5679168

[B36] Mueggler T. Sturchler-Pierrat C. Baumann D. Rausch M. Staufenbiel M. Rudin M. (2002). Compromised hemodynamic response in amyloid precursor protein transgenic mice. *J. Neurosci.* 22 7218–7224. 10.1523/JNEUROSCI.22-16-07218.2002 12177216 PMC6757895

[B37] Ni R. Rudin M. Klohs J. (2018a). Cortical hypoperfusion and reduced cerebral metabolic rate of oxygen in the arcAbeta mouse model of Alzheimer’s disease. *Photoacoustics* 10 38–47. 10.1016/j.pacs.2018.04.001 29682448 PMC5909030

[B38] Ni R. Vaas M. Rudin M. Klohs J. (2018b). “Quantification of amyloid deposits and oxygen extraction fraction in the brain with multispectral optoacoustic imaging in arcAβ mouse model of Alzheimer’s disease,” in *Proceedings of the Conference on Photons Plus Ultrasound: Imaging and Sensing 2018* Vol. 10494 (San Francisco, CA: SPIE BiOS). 10.1117/12.2286309

[B39] Nitsch R. M. Kim C. Growdon J. H. (1998). Vasopressin and bradykinin regulate secretory processing of the amyloid protein precursor of Alzheimer’s disease. *Neurochem. Res.* 23 807–814. 10.1023/A:1022423813362 9566621

[B40] Noda M. Kariura Y. Amano T. Manago Y. Nishikawa K. Aoki S. (2003). Expression and function of bradykinin receptors in microglia. *Life Sci.* 72 1573–1581. 10.1016/S0024-3205(02)02449-9 12551746

[B41] Nokkari A. Abou-El-Hassan H. Mechref Y. Mondello S. Kindy M. S. Jaffa A. A. (2018). Implication of the Kallikrein-Kinin system in neurological disorders: quest for potential biomarkers and mechanisms. *Prog. Neurobiol.* 16 26–50. 10.1016/j.pneurobio.2018.01.003 29355711 PMC6026079

[B42] Passos G. F. Medeiros R. Cheng D. Vasilevko V. Laferla F. M. Cribbs D. H. (2013). The bradykinin B1 receptor regulates Abeta deposition and neuroinflammation in Tg-SwDI mice. *Am. J. Pathol.* 182 1740–1749. 10.1016/j.ajpath.2013.01.021 23470163 PMC3644719

[B43] Paxinos G. Franklin K. (2012). *Paxinos and Franklin’s the Mouse Brain in Stereotaxic Coordinates*, 4th Edn. Cambridge, MA: Academic Press.

[B44] Prediger R. D. Medeiros R. Pandolfo P. Duarte F. S. Passos G. F. Pesquero J. B. (2008). Genetic deletion or antagonism of kinin B(1) and B(2) receptors improves cognitive deficits in a mouse model of Alzheimer’s disease. *Neuroscience* 151 631–643. 10.1016/j.neuroscience.2007.11.009 18191900

[B45] Princz-Kranz F. L. Mueggler T. Knobloch M. Nitsch R. M. Rudin M. (2010). Vascular response to acetazolamide decreases as a function of age in the arcA beta mouse model of cerebral amyloidosis. *Neurobiol. Dis.* 40 284–292. 10.1016/j.nbd.2010.06.002 20600914

[B46] Rodriguez-Vieitez E. Ni R. Gulyas B. Toth M. Haggkvist J. Halldin C. (2015). Astrocytosis precedes amyloid plaque deposition in Alzheimer APPswe transgenic mouse brain: a correlative positron emission tomography and in vitro imaging study. *Eur. J. Nuclic Med. Mol. Imaging* 42 1119–1132. 10.1007/s00259-015-3047-0 25893384 PMC4424277

[B47] Schmaier A. H. (2016). Alzheimer disease is in part a thrombohemorrhagic disorder. *J. Thromb. Haemost.* 14 991–994. 10.1111/jth.13277 26817920

[B48] Spani C. Suter T. Derungs R. Ferretti M. T. Welt T. Wirth F. (2015). Reduced beta-amyloid pathology in an APP transgenic mouse model of Alzheimer’s disease lacking functional B and T cells. *Acta Neuropathol. Commun.* 3:71. 10.1186/s40478-015-0251-x 26558367 PMC4642668

[B49] Stoppe G. Schutze R. Kogler A. Staedt J. Munz D. L. Emrich D. (1995). Cerebrovascular reactivity to acetazolamide in (senile) dementia of Alzheimer’s type: relationship to disease severity. *Dementia* 6 73–82. 7606283 10.1159/000106925

[B50] Tsunoda N. Yoshimura H. (1979). Metabolic fate of noscapine. II. Isolation and identification of novel metabolites produced by C-C bond cleavage. *Xenobiotica* 9 181–187. 10.3109/00498257909038719 473793

[B51] Viel T. Buck H. (2011). Kallikrein-kinin system mediated inflammation in Alzheimer’s disease in vivo. *Curr. Alzheimer Res.* 8 59–66. 10.2174/156720511794604570 21143155

[B52] Viel T. A. Lima Caetano A. Nasello A. G. Lancelotti C. L. Nunes V. A. Araujo M. S. (2008). Increases of kinin B1 and B2 receptors binding sites after brain infusion of amyloid-beta 1-40 peptide in rats. *Neurobiol. Aging* 29 1805–1814. 10.1016/j.neurobiolaging.2007.04.019 17570564

[B53] Vorstrup S. Henriksen L. Paulson O. (1984). Effect of acetazolamide on cerebral blood flow and cerebral metabolic rate for oxygen. *J. Clin. Invest.* 74 1634–1639. 10.1172/JCI111579 6501565 PMC425340

[B54] Zerbi V. Jansen D. Wiesmann M. Fang X. Broersen L. M. Veltien A. (2014). Multinutrient diets improve cerebral perfusion and neuroprotection in a murine model of Alzheimer’s disease. *Neurobiol. Aging* 35 600–613. 10.1016/j.neurobiolaging.2013.09.038 24210253

[B55] Zhang X. Petersen E. T. Ghariq E. De Vis J. B. Webb A. G. Teeuwisse W. M. (2013). In vivo blood T(1) measurements at 1.5 T, 3 T, and 7 T. *Magn. Reson. Med.* 70 1082–1086. 10.1002/mrm.24550 23172845

