# Supplementary material for: fMRI Reveals Mitigation of Cerebrovascular Dysfunction by Bradykinin Receptors 1 and 2 Inhibitor Noscapine in a Mouse Model of Cerebral Amyloidosis
Source: Front Aging Neurosci. 2019 Feb 15;11:27. doi: 10.3389/fnagi.2019.00027 (PMC6413713; doi:10.3389/fnagi.2019.00027)
Supplement: Supplementary file 1 [file Data_Sheet_1.docx]

**
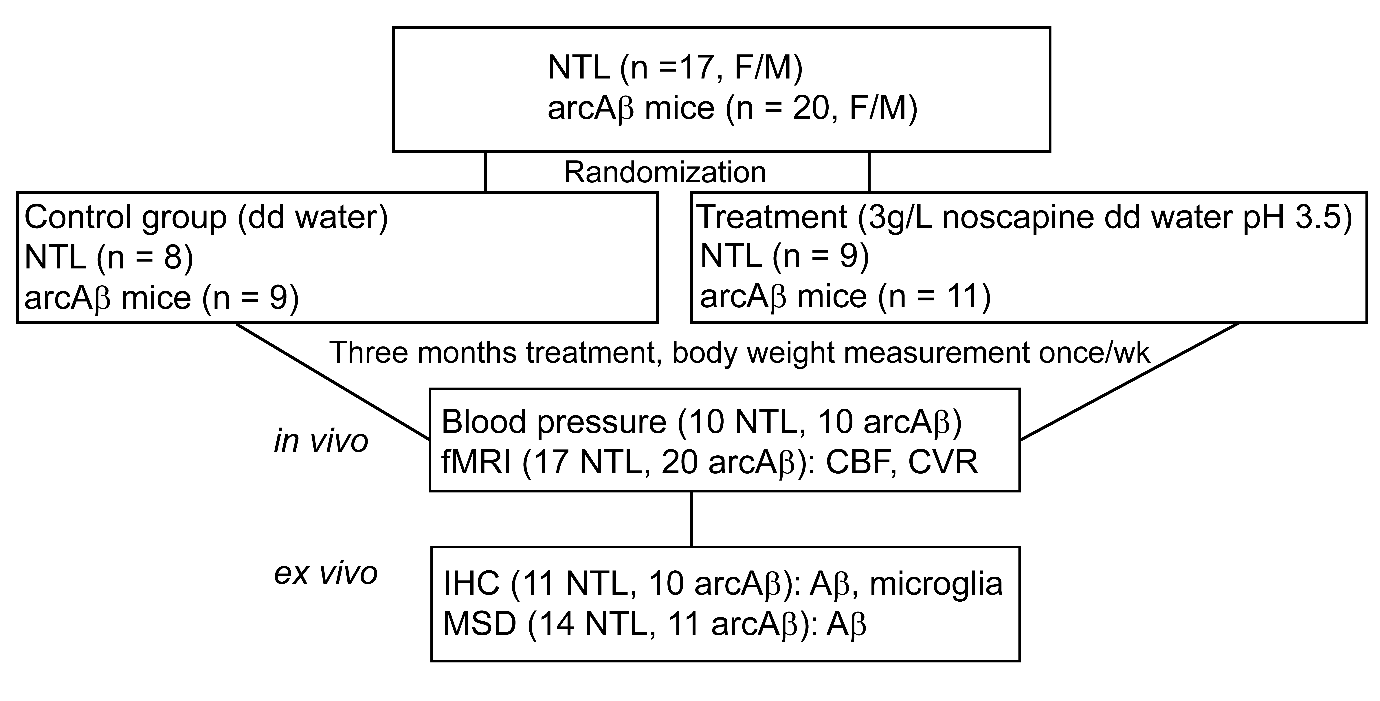
**

**Supplementary Figure 1.** Flowchart of study design.

**
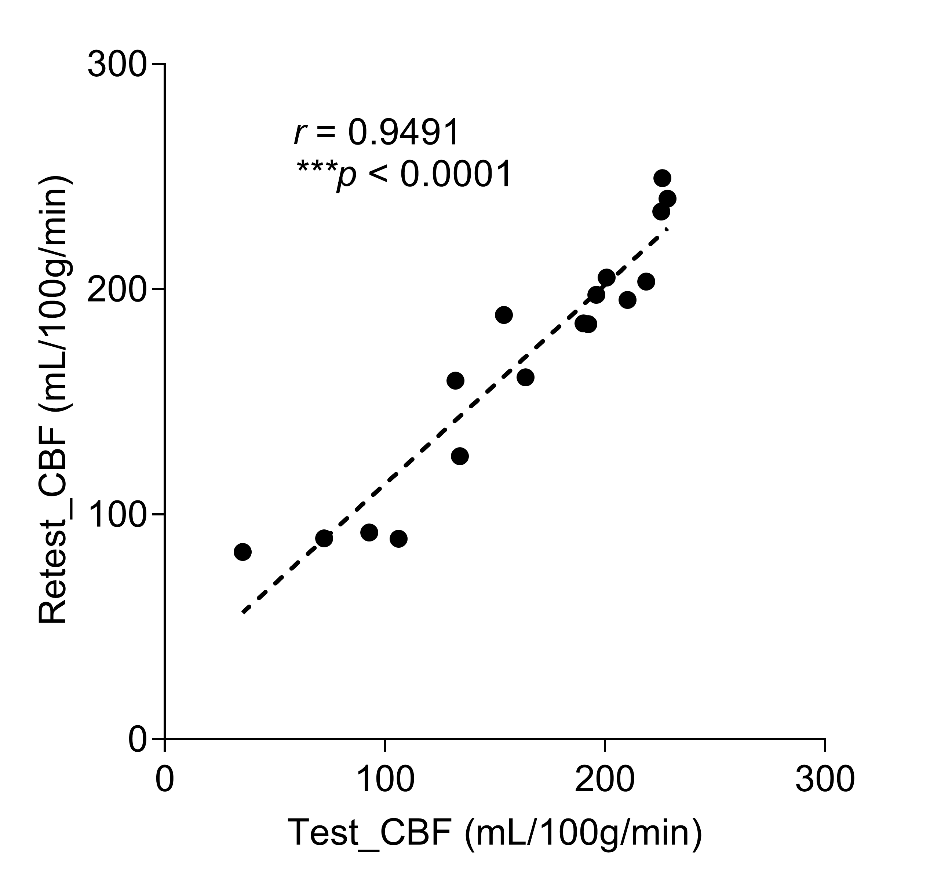
**

**Supplementary Figure 2.** Perfusion MRI shows high test-retest repeatability in quantifying cerebral blood flow (CBF) metrics in mouse brain. Pearson correlation between repeated measurements of CBF, *** *p* < 0.001, n = 17.


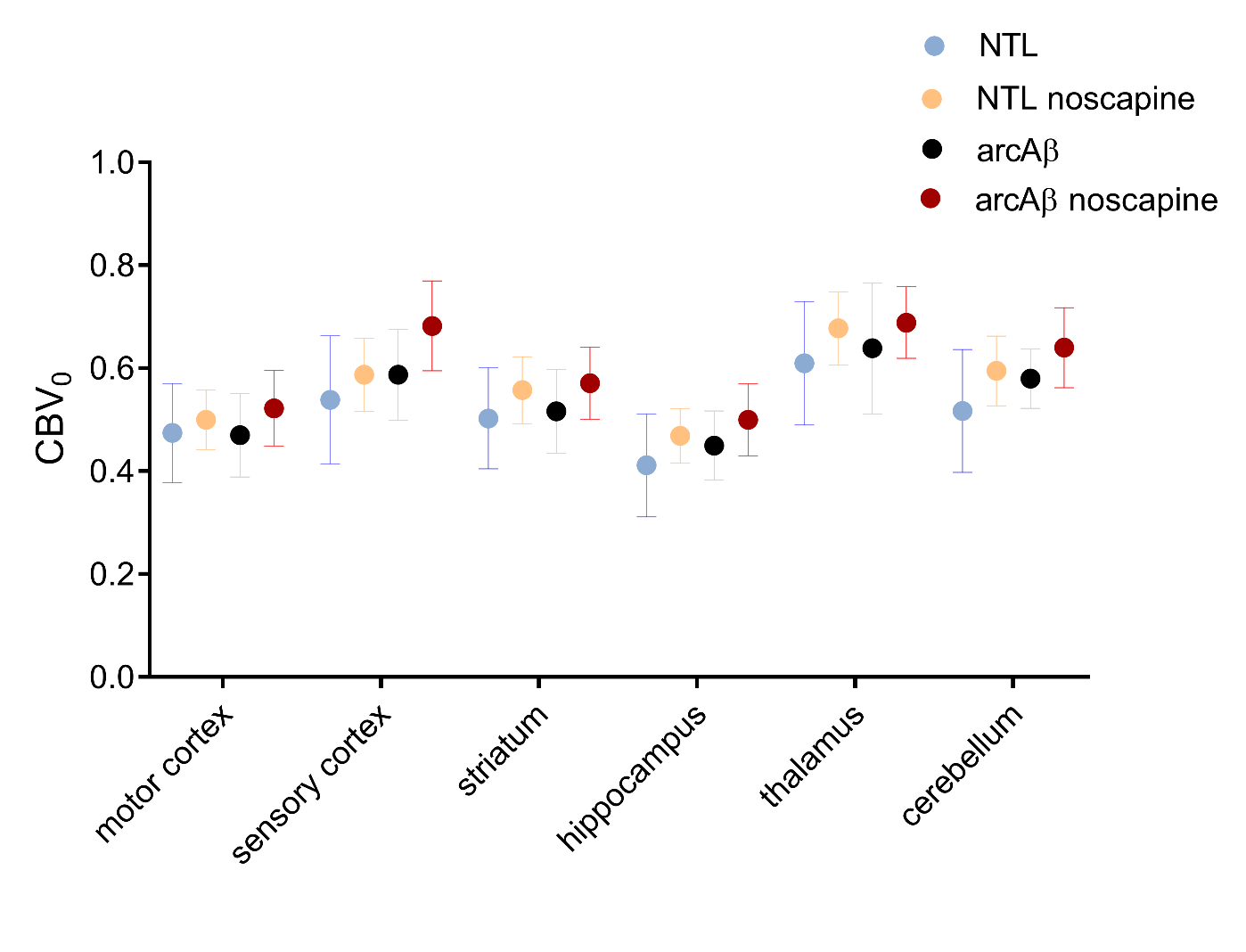


**Supplementary Figure 3.** Quantification of baseline cerebral blood volume (CBV_0_) in brain of the untreated and noscapine-treated non-transgenic littermates (NTL) and arcAβ mice, respectively. Not significant, two-way ANOVA with post hoc Turkey’s correction for multiple comparison; CBF, cerebral blood flow. NTL (n = 8), NTL noscapine (n = 9), arcAβ (n = 9), arcAβ noscapine (n = 11).


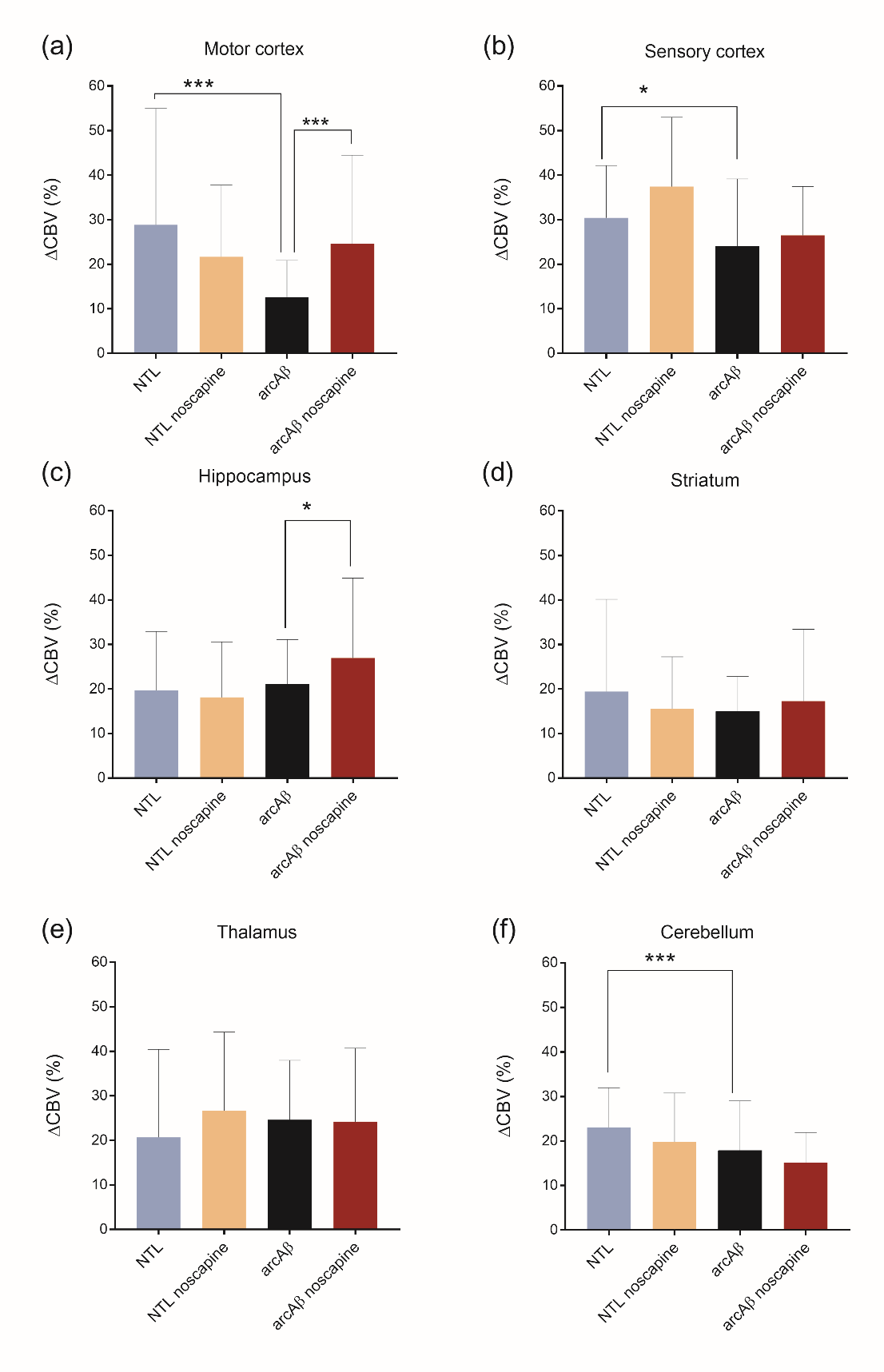


**Supplementary Figure 4.** Quantification of regional cerebral vascular reactivity in mouse brains. **(a-f)** The levels of late ∆CBV (40-46.7 minutes) in six brain regions (motor cortex, sensory cortex, hippocampus, thalamus, striatum and cerebellum) of untreated and noscapine-treated non-transgenic littermates (NTL) and arcAβ mice, respectively; * *p* < 0.05, *** *p* < 0.001, Two-way ANOVA with Turkey *post hoc* multiple comparison; CBV: cerebral blood volume; ∆CBV: percentage change in cerebral blood volume; NTL (n = 8), NTL noscapine (n = 9), arcAβ (n = 9) and arcAβ noscapine (n = 11).
